# Multimaterial actinic spatial control 3D and 4D printing

**DOI:** 10.1038/s41467-019-08639-7

**Published:** 2019-02-15

**Authors:** J. J. Schwartz, A. J. Boydston

**Affiliations:** 1 Department of Chemistry, University of Washington, Seattle, WA 98195 USA; 2 Department of Chemistry, University of Wisconsin, Madison, WI 53706 USA

## Abstract

Production of objects with varied mechanical properties is challenging for current manufacturing methods. Additive manufacturing could make these multimaterial objects possible, but methods able to achieve multimaterial control along all three axes of printing are limited. Here we report a multi-wavelength method of vat photopolymerization that provides chemoselective wavelength-control over material composition utilizing multimaterial actinic spatial control (MASC) during additive manufacturing. The multicomponent photoresins include acrylate- and epoxide-based monomers with corresponding radical and cationic initiators. Under long wavelength (visible) irradiation, preferential curing of acrylate components is observed. Under short wavelength (UV) irradiation, a combination of acrylate and epoxide components are incorporated. This enables production of multimaterial parts containing stiff epoxide networks contrasted against soft hydrogels and organogels. Variation in MASC formulation drastically changes the mechanical properties of printed samples. Samples printed using different MASC formulations have spatially-controlled chemical heterogeneity, mechanical anisotropy, and spatially-controlled swelling that facilitates 4D printing.

## Abstract (web summary)

Objects with varied mechanical properties can be produced by additive manufacturing, but multimaterial control along all three axes of printing is still limited. Here the authors use wavelength control during vat polymerization and demonstrate printing of objects with spatial control of the composition and stiffness.

## Introduction

Additive manufacturing (AM), often referred to as 3D printing, has revolutionized the ability to create advanced parts that display high levels of complexity in their geometry, function, and composition^1–14^. A long-sought capability promised by breakthroughs in AM technology is the rapid production of multimaterial parts. In this regard, abiotic materials production significantly lags the inspiring designs observed in nature. Although new build materials for AM continue to be introduced, the ability to incorporate different types of materials into a single part remains challenging. Typically, multimaterial printing is achieved either through parallel deposition or some form of hybrid manufacturing^10^. Parallel deposition has been demonstrated most prominently through multi-jetting techniques, in which different print heads or nozzles deposit unique photoresins^15–17^. Various forms of material extrusion can also enable multimaterial printing through parallel deposition^11,18–22^. Hybrid manufacturing generally refers to the production of parts by using more than one manufacturing approach. For example, a molded component may be modified by some form of AM technique to give a hybrid part comprised of different materials, or an AM technique could be adapted to allow for incorporation of nontraditional components, such as electronics^23–25^. A notable advance in this area was the advent of EMB3D printing^26–31^. In EMB3D printing, a secondary material is extruded into a primary molded solid host, which has enabling advances in fields such as wearable electronics and soft robotics.

Another exciting approach uses vat photopolymerization combined with clever designs for exchanging the photoresin material during the print^32–40^. This typically involves a method of replacing liquid vat components mid-print. These methods have the advantage of being able to produce hollow voids and overhangs common to vat photopolymerization, and generally do not have the same upper limits on resin viscosity as multi-jetting. However, they typically only achieve heterogeneity along the *z*-axis, whereas control over multiple chemical compositions in the *x*,*y*-plane, and along all three axes, remains challenging.

The methods described above for achieving multimaterial parts with AM offer several advantages and have propelled the field forward in ways that offer creative freedom and innovative capabilities. Inspired by these engineering-based approaches, we considered whether a bottom-up approach based upon chemoselective organic chemistry could offer unique opportunities. Assuming orthogonal chemical reactivities could be achieved with different wavelengths of light in a manner capable of photocuring 2D layers, one would be able to correlate multicolor images with multimaterial compositions. Hawker and coworkers recently developed a similar approach in which they used photochromic dyes to produce a controlled photobleaching front, and thereby a zone of polymerization^14^. Using an acrylate and epoxide-based multimaterial system, they controlled which material polymerized based on wavelength-control over which photochromic dye was photobleached. Importantly, sophisticated macromolecular synthetic methods continue to be discovered and developed, including cutting-edge orthogonal mechanisms mediated by controlled wavelengths of light^41–43^.

Inspired by the potential for fully integrated chemical synthesis and AM technologies, we explore the use of digital light processing AM (DLP-AM) to control chemical composition along all three axes of an object by simultaneously projecting more than one light source into a vat of photoresin. Combining multimaterial and spatial-control DLP-AM (MASC DLP-AM) could provide advantages in multiple area, including stress-focusing designs for mechanoresponsive materials^5,44^, simulated tissue models, controlled gradation in overmolded parts, dynamic optical materials, and autonomous actuators. Herein, we describe our efforts toward using a DLP-based approach for creating multimaterial and spatially controlled compositions as a function of input wavelength.

## Results

### Formulations development

Photoresins for light-based AM processes, including single-laser rastering and DLP-based systems, commonly include a combination of rapidly curable acrylates combined with epoxides that are typically cured during a thermal post-processing step. The resulting homogeneous dual network material benefits first from rapid structure formation, upon curing of the acrylates during printing, and then improved mechanical properties upon thermally setting the epoxide constituents in the post-cure^45^. Notably, acrylate and epoxide curing can be viewed as orthogonal, chemoselective polymerization mechanisms involving radical and cationic intermediates, respectively^46,47^. Considering that each mechanism can be photochemically initiated, we were intrigued by the feasibility of spatially resolving differing acrylate- and epoxide-derived material compositions based upon multiwavelength image projection. Toward this end, we selected Irgacure 819 (reported *λ*_max_ = 295, 370 nm) and a mixture of triarylsulfonium salts (TAS, reported *λ*_max_ = 220, 303 nm) as potential radical and cationic photoinitiators, respectively. For Irgacure 819, we took advantage of the longer wavelength cutoff (*λ*_cutoff_ = 450 nm) in comparison with TAS (*λ*_cutoff_ = 390 nm), as measured in CH_2_Cl_2_ at concentrations relevant to our studies (Supplementary Figure 1). Under visible (white) light irradiation, the Irgacure initiator would be selectively activated and lead to preferential curing of acrylate-based resin components. In contrast, irradiation with 365-nm UV light would also activate TAS and thus was expected to give a higher proportion of epoxide curing via photoacid generation. For acrylate components, we selected 2-hydroxyethyl acrylate (HEA), isobornyl acrylate (IBoA), and butyl acrylate (BA). Each is capable of providing flexible, elastomeric materials upon curing. We note that HEA contains small amounts of diacrylate components that enable crosslinking during DLP-AM^40^. For IBoA-based systems, poly(ethylene glycol) diacrylate (PEGDA, M_n_ 700) was included for crosslinking. For BA-based systems, we included hexanediol diacrylate (HDDA) for crosslinking. Swelling studies were conducted for HEA and BA-based resins. Upon curing, HEA gives cured materials that swell in aqueous media whereas BA-based materials readily swell in organic solvents. These characteristics were expected to be distinct from those of the epoxide-based resin component used in our studies, 3,4-epoxycyclohexylmethyl-3,4-epoxycyclohexane carboxylate (EPOX). Curing of EPOX tends to result in stiff, highly crosslinked materials with low swellability and low flexibility. Empirically, we found that an epoxy-functionalized polyhedral oligomeric silsesquioxane (ePOSS) gave improved printability for IBoA-PEGDA/EPOX-ePOSS (IBoA-1), BA-HDDA/EPOX-ePOSS (BA-1) and HEA/EPOX-ePOSS (HEA-2) resins (Table 1).

**Table 1:** (Top) Idealized representation of using the HEA-1 MASC formulation to achieve different materials with different wavelengths of light. (Bottom) MASC formulations used in this study

				
Formulation	Acrylate monomers (%)^a^	Epoxide monomers (%)^a^	Photoinitiators (wt %)^b^	Additives (wt %)^b^
HEA-1	HEA (30)^c^	EPOX (70)	Irgacure 819 (0.4), TAS (8)^e^	hydroquinone (0.12)
HEA-2	HEA (50)^c^	EPOX (46.5), ePOSS (3.5)^d^	Irgacure 819 (0.4), TAS (5)^e^	hydroquinone (0.12) Nile Red (0.005)
IBoA-1	IBoA (45), PEGDA (5)	EPOX (46.5), ePOSS (3.5)^d^	Irgacure 819 (0.4), TAS (5)^e^	hydroquinone (0.12)
BA-1	BA (28.75), HDDA (1.25)	EPOX (56), ePOSS (14)^d^	Irgacure 819 (0.4), TAS (5)^e^	hydroquinone (0.12)

^a^Percent by weight of total monomers

^b^Weight percent based on weight of total monomers

^c^Stock HEA solution contains 9 wt % oligo(ethylene glycol) diacrylates as determined by GC-MS (*40*)

^d^Estimate reflects commercial composition of ePOSS, which is reported to contain 30% EPOX by weight

^e^Triarylsulfonium salts obtained commercially as a 50% solution (by wt) in propylene carbonate and used as-received

In general, bulk cure times with white light and HEA/Irgacure were shorter than those for UV light and EPOX/TAS combinations. To compensate for this difference, we screened varied ratios of the components and found that a 3:7 ratio of the acrylate- versus epoxide- monomers provided serviceable cure times (<120 s) using our custom visible and 365-nm UV projector sources (Supplementary Figure 2). Incorporation of ePOSS in the IBoA-1 and HEA-2 system enabled a 1:1 ratio of radical to cationic components. Additionally, we used 0.4 wt % of the Irgacure 819 initiator versus 2.5–4 wt % of the TAS.

For the EPOX/TAS curing, we screened a series of layer cure times at an average maximum UV power density of 0.75 mW/cm^2^, which was the highest value achievable with our current optical setup. Layer cure times of less than 1 min per layer resulted in incomplete fixity of the material, whereas layer cure times of >2 min caused lamination issues between the specimen and the vat lining. Therefore, layer cure times of 1- and 2-min were used in our studies. Printing with visible light was found to be straightforward and matching the 1–2 min layer cure times was easy to achieve. Modification of the formulation with regard to initiator concentration was met with limited success in reducing layer cure times. We anticipate that higher intensity of UV light or increased initiator efficiency could be successful in future iterations. Gel fractions were determined as described below in the methods section, and indicated that printing with UV light cured roughly twice as much material as when printing with visible light (Supplementary Table 1). Homogeneous samples printed with either UV or visible light were also analyzed by cross-polarization magic angle spinning (CP-MAS) NMR spectroscopy (Supplementary Figures 4–10). Qualitative assessments of the CP-MAS NMR spectra were consistent with selective polymerization of acrylate species under visible light irradiation. Curing with UV light led to samples having peak resonances consistent with both acrylate and epoxide polymerization. These results signified the general ability to dictate the chemical compositions of parts printed from a single vat using simple wavelength control.

### Printing and multimaterial characterization

**Fig. 1:**
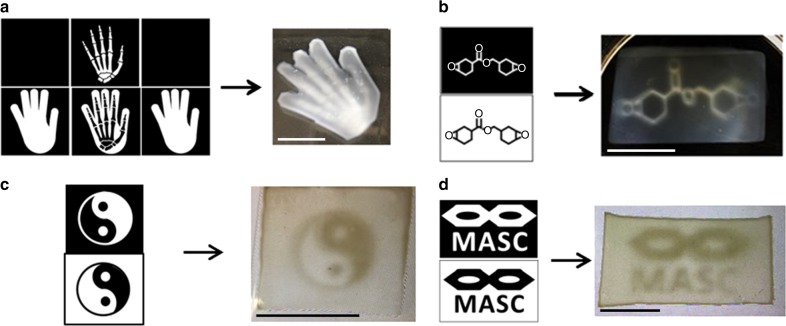
Representative sets of DLP projections (arbitrary scaling) for UV (top images) and visible light (bottom images), and their corresponding printed specimens. All samples were printed using the BA-1 MASC formulation. Scale bars correspond to 25 mm. **a** A 3D design resembling a hand with encased internal bones. Picture taken after being removed from toluene. **b** An EPOX molecular structure signifying the opaque region containing the EPOX-based material. Picture taken on a black background in toluene. **c** A yin yang design. Picture taken with backlighting in toluene. **d** An in-house logo for our MASC printing process. Picture taken with backlighting in spearmint oil

To enable multimaterial DLP-AM using the MASC formulations, we created binary image combinations that would combine to form a multimaterial printed part. One series of images was processed through a visible-light DLP projector while the other was sent in parallel to a custom-built UV-projector system (Supplementary Figure 2). Select examples of multimaterial printed specimens are depicted in Fig. 1. The contrast between the different regions was clear to see when samples printed with the BA-1 were submerged in toluene or spearmint oil. The more optically clear regions correspond to segments printed with visible light, whereas the opaque segments were produced with UV light. Three of the examples have hard regions that run fully through each specimen along the *z*-axis (Fig. 1b–d). The first example, which resembles a hand with interior bones, has hard segments that are fully encased within the softer continuous phase (Fig. 1a). These samples collectively demonstrate the unique ability to easily control multimaterial composition along all three axes of printing.

**Fig. 2:**
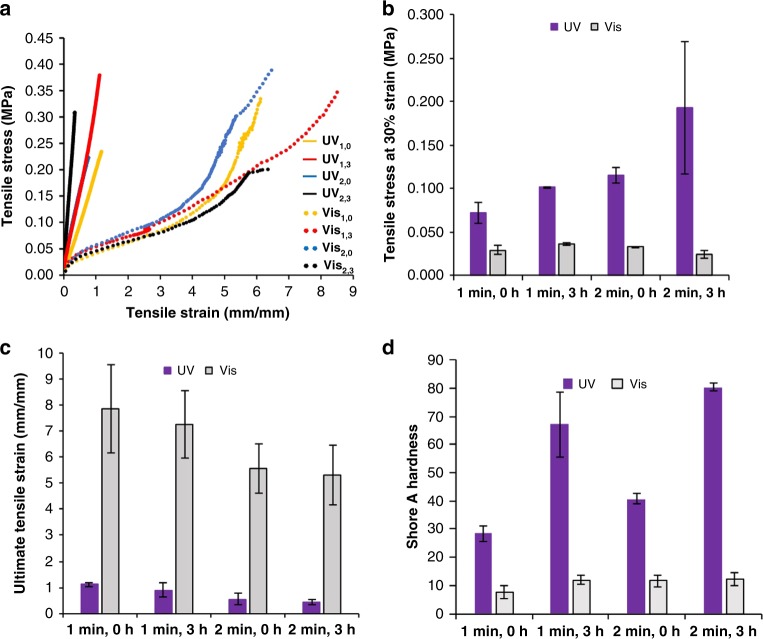
Representative stress-strain plot and data from tensile testing of HEA-1 samples. **a** Representative stress–strain plots for uniaxial tensile test HEA-1 specimens each printed with only one light source. To denote each specimen type, we use the light source (UV or Vis) and subscript numbers to indicate the layer cure time in minutes and the thermal post-cure time in hours. For example, UV_1,3_ samples were produced using UV light with 1-min layer cure times and a 3-h thermal post-cure. **b** Comparison of relative stiffness of samples at 30% strain; samples printed with UV (purple) or visible light (light gray) from a single vat. **c** Comparison of ultimate tensile strain of samples printed with UV (purple) or visible light (light gray) from a single vat. **d** Comparison of Shore A hardness values; samples printed with UV (purple) or visible light (light gray) from a single vat. All error bars correspond to one standard deviation

Having established acceptable conditions for layer curing and achieving controlled materials compositions from the MASC formulations, we next characterized the mechanical properties resulting from printing selectively with either visible or UV light. Tensile test specimens were printed from the HEA-1 system using either 1- or 2-min layer cure times. Additionally, we examined the effects of thermal post-processing at 60 °C, which was found to increase the stiffness of materials printed with UV light but not those printed with visible light. This is consistent with as-printed samples having residual (unreacted) epoxide functional groups that further react in the thermal treatment, ultimately increasing crosslink density in the material. As expected, the light source used during printing, and therefore the chemical compositions of the test bars, dominated the outcomes of the stress–strain behavior (Fig. 2). Printing with UV light gave stiff specimens with linear elastic stress–strain behavior prior to fracture. In contrast, printing with visible light led to much more compliant parts that displayed viscoelastic behavior. Independently, increased layer cure times and thermal post-curing each led to higher modulus material when printing with UV light.

The differences in relative stiffnesses are further elaborated in Fig. 2b, c. With increased layer cure time and thermal processing of the samples printed with UV light, we observed a gradual increase in the stress at 30% tensile strain (Fig. 2b), ranging from 0.07 to 0.19 MPa. The soft samples, printed using visible light, had much lower values of stress at 30% strain, ranging from 0.02 to 0.04 MPa. Ultimate tensile strain was also found to be significantly different between samples printed with visible versus UV light (Fig. 2c). For example, the lowest ultimate tensile strain for the soft specimens was ca. 570%, whereas the highest value observed for the stiffer specimens was ca. 111%. Finally, we measured the Shore A hardness of the different samples (Fig. 2d).

**Fig. 3:**
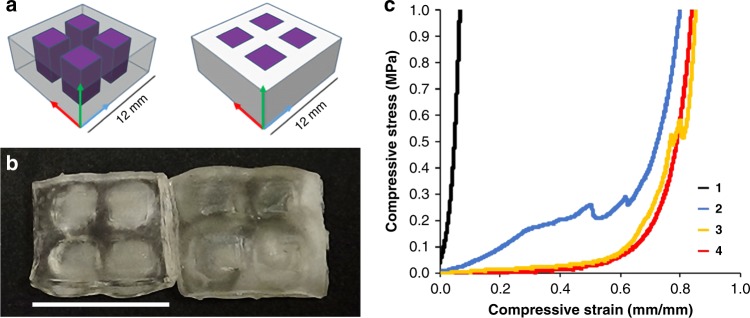
Design, print, and representative stress-strain plot from compression testing of multimaterial HEA-1 printed specimens. **a** CAD models of 4-pillar multimaterial objects and representative printed specimens. Purple corresponds to pillars printed with UV light and white/transparent outer region corresponds to components printed with visible light. Arrows denote axis of printing: *x*-axis (blue), *y*-axis (red), *z*-axis (green). **b** Printed specimens: (left) no thermal post-cure; (right) 3-h thermal post-cure at 60 °C; each using 1-min layer cure times. As-printed outer object dimensions: 12 × 12 × 5 mm^3^. Pillar dimensions: 3 × 3 × 5 mm^3^ with 2-mm spacing. Scale bar corresponds to 12 mm. **c** Representative compressive stress-strain plots of HEA-1 specimens. All boxes printed with 1-min layer cure times and post-cured for 3 h at 60 °C. Black (1) = homogeneous sample cured with UV light. Blue (2) = pillar box sample compressed along the *z*-axis. Yellow (3) = pillar box sample compressed along the *x*-axis. Red (4) = homogeneous sample cured with visible light

Excited by these results, we moved to larger, more complex mechanical samples. Toward this end, we designed models for uniaxial compression tests that consisted of stiff internal pillars surround by a soft outer continuous phase (Fig. 3). With the first design, we prepared 4-pillar specimens using the HEA-1 MASC formulation. For clarity, 3D CAD models are depicted in Fig. 3a along with representative printed specimens (Fig. 3b). Homogeneous specimens were also printed using either UV or visible light.

Uniaxial compression (Fig. 3c) showed homogeneous samples printed with UV light to again be stiffer than those printed with visible light. Specifically, printing with UV light produced samples that reached the limit of the 500-N load cell at ca. 10% compressive strain (Fig. 3c, black). Increasing to a 250-kN load frame revealed an average onset of fracture at 19% compressive strain, which occurred at an average stress of 28 MPa (Supplementary Figure 11). The softer samples printed with only visible light (Fig. 3c, red) underwent much greater compressive strains of ca. 90% without reaching the 500-N load limit, and no visible onset of fracture was observed. The multimaterial specimens displayed anisotropic compressive behavior, and stress–strain curves that were significantly different from their homogeneous counterparts. Compression of the 4-pillar samples perpendicular to the long axis of the support pillars (compression along the *x*-axis) gave stress–strain curves that initially overlapped those of the homogeneous soft samples (Fig. 3c, yellow). However, at ca. 59% strain, compaction of the sample likely caused stress to be distributed more evenly across both the soft and hard segments, resulting in an apparent stiffening of the specimen. At ca. 75% strain and above, we observed features in the stress–strain curves that were ascribed to fracturing of the internal stiff pillars. These failure events were confirmed visually upon inspection of the samples after compression. Compression of the 4-pillar samples along the *z*-axis, parallel to the long axis of the pillars, led to an earlier onset and greater extent of apparent stiffening (Fig. 3c, blue). In this case, the initial stiffness was intermediate between the homogeneous soft and hard specimens and had an apparent bilinear behavior until ca. 50% strain. Above 50% compressive strain, we again observed features that were consistent with failure of the internal support pillars. We also investigated compression of multimaterial 2-pillar design samples based upon the BA-1 MASC formulation, and found the results to be similar (see Supplementary Information, Supplementary Figure 12).

**Fig. 4:**
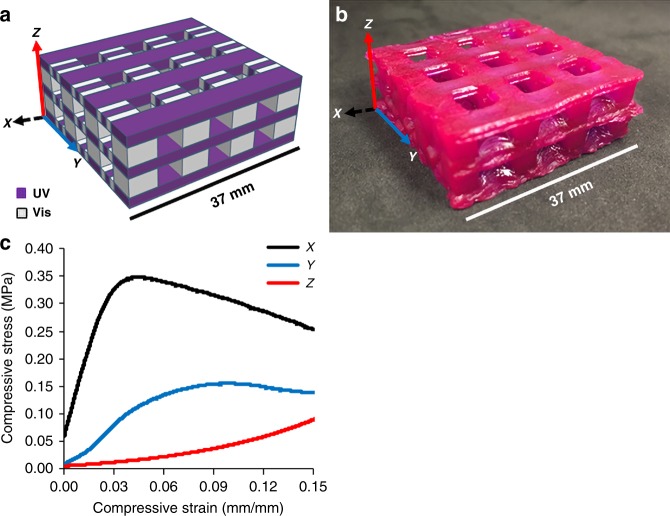
Design, print, and representative stress–strain plot from compression testing of multimaterial HEA-2 printed specimen. **a** CAD model of tetragonal lattice. Purple corresponds to regions printed with UV light and white corresponds to regions printed with visible light. **b** Printed tetragonal lattice using HEA-2 MASC formulation. Sample printed with 1-min layer cure times, and then thermally post-processed at 100 °C for 10 min. Sample dimensions: 37 × 37 × 13 mm^3^. Nile Red was used as a dye to reduce visible light-induced outgrowth. **c** Representative stress-strain plots of compression along all three axes. Black = *x*-axis with stiff beams printed with UV light. Blue = *y*-axis with multimaterial beams printed with both UV and visible light. Red = *z*-axis dominated by soft beams printed with visible light

We next used a MASC-DLP formulation to demonstrate how mechanical anisotropy could be achieved in a complex lattice framework. More commonly, geometric variation using monolithic materials is used to create different mechanical responses as a function of the axis of loading^48^. Herein, we used compositional variation within a lattice of high geometric symmetry. We designed a tetragonal lattice that had different mechanical properties along all three axes of printing (Fig. 4a, b). For this design we found HEA-2 to be successful. In our tetragonal lattice design, the *x*-axis beams were printed with UV light to provide stiff rows. The *y*-axis had a mixture of regions printed with either UV or visible light to provide a composite response. The *z*-axis was prepared predominately with visible light, to provide a less stiff, viscoelastic response under compression. In compression along the *x*-axis (Fig. 4c, black), a clear linear elastic response was observed, dominated by the incorporated epoxide-based material. The modulus of the lattice in the *x*-axis was found to be 10.4 MPa. Compression along the *y*-axis (Fig. 4c, blue) showed a bilinear response up to 3% compressive strain. Compression along the *z*-axis exhibited a less stiff, viscoelastic response (Fig. 4c, red), suggesting the modulus of the lattice along this axis is strain-rate dependent. Comparison of the relative stiffnesses at 3% strain showed the lattice to be 4.06 and 26.2 times stiffer in the *x*-axis than the *y*- and *z*-axes, respectively. Once again, this object demonstrates the ability to control material composition and mechanical properties along all three axes of printing.

Modulation of physicochemical properties was also possible by varying the composition of the MASC-DLP formulations. For example, the HEA-1 and HEA-2 formulations give relatively soft materials for the acrylate-dominated regions printed with visible light. These materials while low stiffness, can provide very high elongation elastomers (600–800% elongation). To increase stiffness, we exchanged the HEA for isobornyl acrylate (IBoA) and included polyethylene glycol diacrylate (PEGDA) as a crosslinker for the acrylate portion, along with a 9:1 EPOX/ePOSS mixture for the epoxide portion (Table 1, IBoA-1). Analysis of tensile test specimens as before revealed a ca. 65-fold increase in elastic modulus for the samples printed with visible light when going from HEA-1 to IBoA-1 (elastic modulus = 0.16 and 10 MPa, respectively, Supplementary Figures 13 and 14). Comparison of samples printed using UV light revealed a large increase in elastic modulus for HEA-1 versus IBoA-1 (ca. 260-fold increase). This larger increase in elastic modulus results from the addition of ePOSS to the epoxide component of the MASC formulation^49^. The materials produced from IBoA-1 have a much higher overall stiffness, and toughness, but much lower maximum elongation (~130% elongation). The increase in elastic modulus when printing with UV light relative to visible light is consistent with the properties of the dual network composition being dependent upon both the acrylate and epoxide monomer types. Measured hardnesses of IBoA-1 samples were also found to be much higher than those of HEA-1 samples (Supplementary Figure 13). These variations in the photoresin formulations demonstrate the ability to adapt and modify the multicomponent compositions toward specific material properties and desired applications.

### 4D printing

We also investigated the swelling behavior of objects printed with the MASC-DLP formulations, with the aim of creating multimaterial actuators (i.e., 4D printing). As expected, specimens that were printed with visible light to give predominantly HEA or BA compositions swelled in aqueous and organic solvents, respectively (Supplementary Table 2). In contrast, specimens printed with UV light to incorporate EPOX showed considerably less volumetric swelling. Specimens printed with sequential UV and visible light resulted in intermediate swelling ratios. Specifically, the mass change in deionized water for HEA-1 samples printed with visible light was found to be 2.2 times that of samples printed using UV light. By volume, the ratio between the two was even greater, with samples printed with visible light swelling 4.6-fold more than those printed with UV light. Notably, thermal post-curing (without prior solvent extraction) reduced the swelling of samples printed with UV light, whether homogeneous or multimaterial compositions (Supplementary Table 3). In toluene, BA-1 samples printed with visible light swelled 1.7 times more by mass, and 2.0 times more by volume, than samples printed with UV light.

**Fig. 5:**
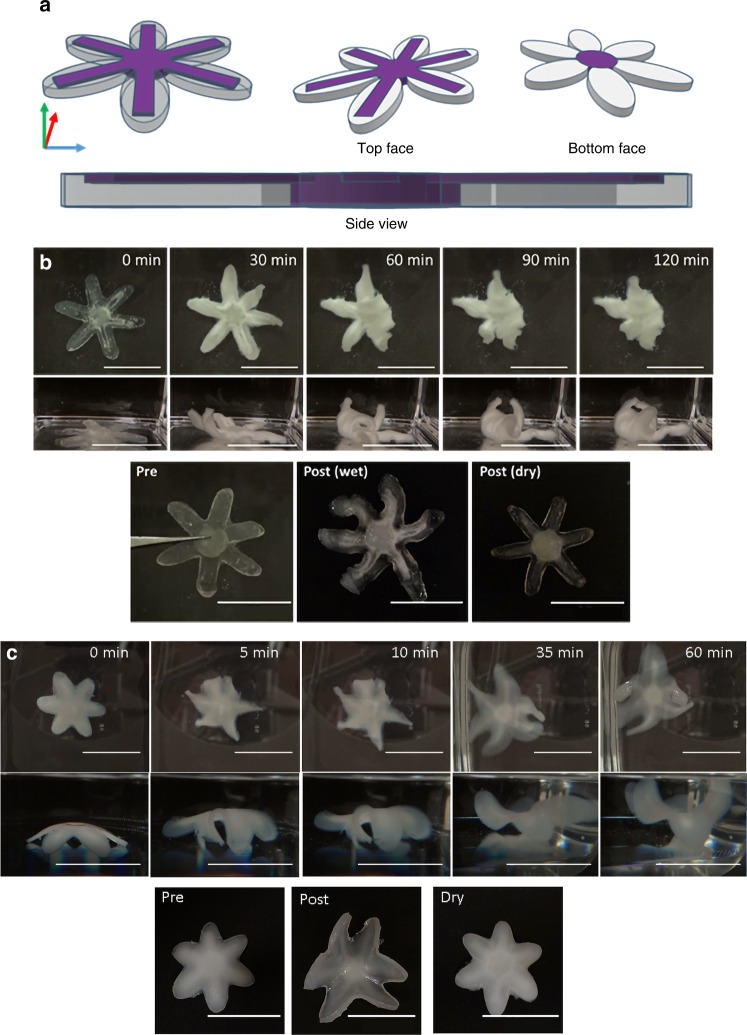
Time-lapse photos of swelling induced actuation in printed sea stars. **a** CAD models of multimaterial sea star. Tip-to-tip length = 38 mm, core diameter = 9 mm, inlaid beams within each arm = 13 mm. Purple corresponds to UV irradiation and white/transparent corresponds to visible light irradiation. **b** Swelling results of a sea star in water printed using the HEA-1 MASC formulation. Scale bars = 25 mm. **c** Swelling results of a sea star in toluene printed using the BA-1 MASC formulation. Scale bars = 25 mm

To investigate 4D printing applications^9^, we prepared multimaterial objects that were designed to undergo actuation upon spatially-controlled swelling. We expected that components produced with UV light would function as strain-limiting regions that guide actuation. To explore, we designed a sea star comprised of a stiff center region and multicomponent arms (Fig. 5a). The arms were constructed with narrow hard segments along the top center axis of each.

We first printed the sea star using the HEA-1 MASC system and observed the results of swelling in deionized water (Fig. 5b, Supplementary Movie 1, left). The sea star arms consistently curled upward, toward the strain-limiting stiff regions along the top of each arm. Across several samples, the curling of the arms happened sequentially without any obvious predictability to the pattern (i.e., it appeared to be stochastic and unrelated to printing orientation). The BA-1 MASC system also enabled 4D printing through swelling-induced actuation (Fig. 5c, Supplementary Movie 1, right). The acrylate components swelled to greater extent in toluene than did the epoxy-based regions, again leading to curling of the sea star arms. Unlike the HEA-1 samples, which quickly curled toward the strain-limiting inlaid UV cured beams, the BA-1 sea star seemed to first curl away from the beams. With continued swelling, the arms then lifted upwards to give a final shape consistent with the design.

## Discussion

The work described herein establishes a method for multimaterial AM that is inspired by bottom-up chemical approaches to materials synthesis. By correlating light inputs from DLP projection with chemical composition, MASC-AM enables one to rapidly dictate spatially-controlled heterogeneity throughout the entire volume of a printed part. The disparate materials combinations have manifested heterogeneous imagery, mechanical anisotropy, and 4D printing through spatially controlled swelling, each from single resin vats simply by inputting controlled combinations of light. This is a first step toward an expanded capability that we hope empowers designers, artists, engineers, and scientists to push the limits of AM materials combinations. Although these first demonstrations focused on a select combination of materials, expansion toward including the literal and figurative spectrum of photochemical reactivity could unlock numerous possibilities for multimaterial AM.

## Methods

### General considerations

2-hydroxyethyl acrylate (HEA), 3,4-epoxycyclohexylmethyl-3,4-epoxycyclohexane carboxylate (EPOX), butyl acrylate (BA), hexanediol diacrylate (technical grade, 80%) (HDDA), poly(ethylene glycol) diacrylate (PEGDA) (M_n_ 700), triarylsulfonium hexafluoroantimonate salts (50% in propylene carbonate) (TAS), Irgacure 819, and hydroquinone were purchased from Sigma-Aldrich and used without further purification. Isobornyl acrylate (IBoA) ( > 90% purity) was purchased from TCI Chemicals and used without further purification. An Epoxycyclohexyl POSS Cage Mixture (ePOSS) (EP0408 formulation EP3F08.04: 70 wt % POSS and 30 wt % EPOX) was purchased from Hybrid Plastics and used without further purification. A LittleRP was used as the printer with levelling modifications to the build plate that provided the ability to use 3-point levelling to modify the angle of build plate to match the build vat^11^. A DLP 6500 LightCrafter kit with a fiber lightguide adapter was purchased from DLinnovations and modified to include quartz optics. An UHP-T-365-LA 365 nm LED, controller, and 3 mm core light guide was purchased from Prizmatix and connected to the projector as visible in Supplementary Figure 2. A FGUV11 filter was included with the LED and light guide to remove residual violet and visible light from the 365 nm light source. Combining the LED light source with the LightCrafter results in a 365 nm DLP projector. Using a SPER Scientific UVA/B light meter 850009, the average light intensity of UV light at the build plate was 0.75 mW/cm^2^. An Optoma HD27 1080p DLP Home projector was used with standard Vivid projection settings for the visible light projector. Using an Extech HD450 Light meter at the build vat of the printer recorded an average light intensity of 80 klx. The projectors were connected to and controlled by a Dell Inspiron 3531 laptop using a StarTech Triple-Monitor USB 3.0 Docking Station. The photoresin vat was made in-house to include a 3 mm quartz plate bottom surface for printing and was lined with approximately 11 g of Sylgard 184 (10:1 ratio of base to curing agent by mass). Hardness measurements were made using a digital Shore A or Shore D durometer.

### Tensile and compressive testing

Tensile and compressive testing were done in duplicate on a Instron 5585 H Universal Testing System equipped with BlueHill 3 software. Tensile testing was done according ASTM D638 using type V specimens using a 50-N (all HEA-1 samples, and IBoA-1 samples cured with visible light) or 2-kN (IBoA-1 samples cured with UV) load cell with pneumatic or 1-in. static wedge grips, respectively. Elongation was conducted at a 10 mm/min extension rate and an Instron 2663–821 Advanced Video Extensometer was used to track strain in HEA-1 and IBoA-1 samples. Compressive testing was done using a 500-N, 1-kN or 250-kN load cell at a rate of 0.5 or 1 mm/min depending on the resin (0.5 mm/min for HEA-1 (500-N, 250-kN), 1 mm/min for BA-1 (500-N) and HEA-2 (1-kN)).

### CP-MAS solid-state NMR spectroscopy

For cross-polarization magic angle spinning solid-state NMR spectroscopy, all samples were packed with magnesium oxide to improve sample balance and spin. Solid-state NMR experiments analyzing HEA-1 samples were conducted using a 16.4 T magnetic field (^1^H resonant field of 700.18 MHz) on a Bruker Avance III spectrometer fitted with a ^1^H{^31^P, ^13^C} 3.2 mm MAS probe. Solid-state NMR experiments analyzing IBoA-1 samples were conducted using a 11.75 T magnetic field (^1^H resonant field of 500.22 MHz) on a Bruker Avance III spectrometer fitted with a 4 mm ^1^H/X/Y DSI MAS probe. A magic angle spinning rate of 12.5 or 12 kHz was used for HEA-1 or IBoA-1 samples, respectively. The ^13^C NMR signal was enhanced using cross-polarization (CP) with a ^1^H–^13^C contact time of 1.2 ms. With a recycle delay of 3 s, the CP-MAS experiments were performed with a proton 90° pulse time of 4 or 4.55 µs for HEA-1 or IBoA-1 samples, respectively. For the HEA-1 samples, the number of scans was 32,768, with the number of points collected for the experiments was 1024, and a sweep width of 598 ppm. For the IBoA-1 samples, 2400 to 4000 scans were acquired and the acquisition time was 30 ms, with 3742 points collected for a sweep width of 497 ppm. All shifts reported were referenced to tetramethylsilane (TMS) in the solid state indirectly using adamantane (*δ* *=* 38.48)^50–52^.

### Resin formulation

Resin formulations were stored in the dark and generally used within one week of preparation. Hydroquinone, Irgacure 819, and TAS were each added into a screw-cap jar. Then, acrylate and epoxide monomers were added. The jar was then sealed with a screw cap, covered with aluminum foil, and then the mixture was manually shaken until complete dissolution was visually observed. Nile Red, when added, is mixed into the resin directly before printing. Resin formulations are provided in Table 1.

### DLP 6500 LightCrafter modification

Although marketed as a UV printer, the DLP 6500 LightCrafter does not have UV-grade quartz lenses. As such, all lenses were removed from the LightCrafter and replaced with a simple UV-grade quartz setup. A general diagram of the lenses used (two collimating lenses prior to the DMD chip, and one projection lens) and UV projector can be found in Supplementary Figure 2.

### Projector alignment and calibration

PowerPoint 2016 was used to identify overlapping regions for the two projectors, and then these areas were used for all further prints (Supplementary Figure 3). Both projectors have a resolution of 1920 × 1080 pixels. The Optoma HD27, being a home projector system, has a much larger projected image/ throw ratio than our modified LightCrafter, and so a much smaller area of a 1920 × 1080 pixel image is used. The pixel size for the UV projected image is the total 1920 × 1080 area, and the pixel size of the corresponding visible light image 648 × 365. With the digital areas and locations identified, image processing for the two projectors became easy, as any image displayed on the UV projector could be displayed on the visible projector, by reducing its size by 33% and placing it in a PowerPoint slide in its proper location. In terms of physical alignment, the UV projector was locked into place on a breadboard such that nothing could move except for the two collimating lenses the project the light guide onto the DMD chip. With a white image displayed on the DMD chip, the intensity of the UV at the build plate was measured using a light meter. If the light intensity was not averaging 0.75 mW/cm^2^, then the collimating lenses were adjusted until the image was uniform that the intensity correct. The Optoma HD27, our visible light source, is placed to the side of the UV LightCrafter and projects at a slight angle. To account for this angle, digital images are rotated 7 degrees. An example processed image can be found in Supplementary Figure 3. The visible projector alignment and light intensity is checked in relation to the UV projected image before every printing session.

### Printing with MASC formulations

A modified stereo mode version of Creation Workshop 1.0.0.55 was used to send sliced images to both projectors simultaneously, identifying individual left (visible) and right (UV) projectors. Images for printing were made using PowerPoint 2016 and resized to 1920 × 1080 pixel images in MSPaint. A modified stereo version of Honeyguide 0.13 was used to incorporate images into a Creation Workshop Scene file (.CWS) with the desired print/slice settings and assign them to either the left (visible) or right (UV) projector. Once images were assigned to projectors in the slice file and the program connected to both projectors, prints would be run in an identical manner to when a single projector was used. Print settings used: layer cure time, 60,000 ms or 120,000 ms; layer thickness, 0.1 mm; Z lift distance, 6–8 mm; Z lift speed, 100 mm/min; Z retract speed, 100 mm/min; blanking layer time, 20,000 ms. The gcode of the.CWS was modified such that during the blanking layer the build plate retracts, waits ten seconds, returns to the correct build vat depth, and then waits another 10s (20 s total) before starting the next layer. This was done to help with adhesion issues.

### Post-processing

After printing the objects, HEA-1 samples were either heated in a 60 °C oven for 1, 3, or 5 h or immediately quenched in solvent. Heating can be used to increase the extent of curing of the cationic polymerization but can result in a loss in multi-material resolution. HEA-2 lattice designs were lightly washed with acetone and then placed in an oven at 100 °C for 10 min. Heating was not deemed necessary for BA-1 samples in this study. After printing or heating, the living epoxide polymerization was quenched by swelling the object in acetone. HEA-1 objects comprising 10–20 layers were repeatedly swelled in deionized water and then soaked in an acetone wash for 30 min–1 h each (×3) to remove unreacted monomer. Thicker HEA-1 and HEA-2 objects, 30–142 layers, required more repeated swellings in acetone, as many as 8. In cases where large amounts of swelling in water could damage the object geometry, CH_2_Cl_2_ was used instead. For BA-1 samples, CH_2_Cl_2_ was used to remove monomer by swelling for 30 min (×3). IBoA-1 samples (comprised of 32 layers) were swelled in acetone for 15 min (×5) to remove unreacted monomer. Deswelling of IBoA-1 samples was done slowly under an inverted beaker to provide some vapor pressure and prevent fast-shrinkage induced defects. Monomer was considered removed when the mass of the dry object before and after swelling was within 3% difference.

### Determination of gel fractions

Printed discs were wiped with acetone after removal from the vat and weighed. Samples were then placed individually in a Supelco small Soxhlet extraction apparatus (50 mL extractor capacity, extractor I.D. 30 mm, 125 mL flask capacity, glass thimble), and successive extractions were carried out over 6 h with CH_2_Cl_2_ for HEA-1 samples, and over 3 h with CH_2_Cl_2_ for BA-1 and IBoA-1 samples. Dried samples after extraction were also weighed, and gel fraction calculated.

### Swelling studies

Printed cylinders comprising of ten layers were swelled in deionized water (3 h) or toluene (2 h) after initial workup and monomer removal. Tests were done in triplicate. The volume and mass of the cylinders were recorded before swelling, immediately after swelling, and again when dry. Percent swelling by mass and volume were calculated using the following equation:

1 $${\mathrm{Swelling}}\left( {\mathrm{\% }} \right) = \frac{{S - D}}{D} \times 100$$

Where *S* is the volume or mass of the swelled material, and *D* is the volume or mass of the post-swelling dry material, respectively.

### Reporting summary

Further information on experimental design is available in the Nature Research Reporting Summary linked to this article.

## Supplementary information

Supplementary Information

Description of Additional Supplementary Files

Supplementary Movie 1

Reporting Summary

## Data Availability

A copy of the software used for printing in this study is available upon request through the corresponding author email.
