# Supplementary material for: Multimaterial actinic spatial control 3D and 4D printing
Source: Nat Commun. 2019 Feb 15;10:791. doi: 10.1038/s41467-019-08639-7 (PMC6377643; doi:10.1038/s41467-019-08639-7)
Supplement: Supplementary file 1 — Supplementary Information [file 41467_2019_8639_MOESM1_ESM.pdf]

Supplementary Information

Multimaterial Actinic Spatial Control 3D and 4D Printing

*Johanna J. Schwartz, and Andrew J. Boydston**

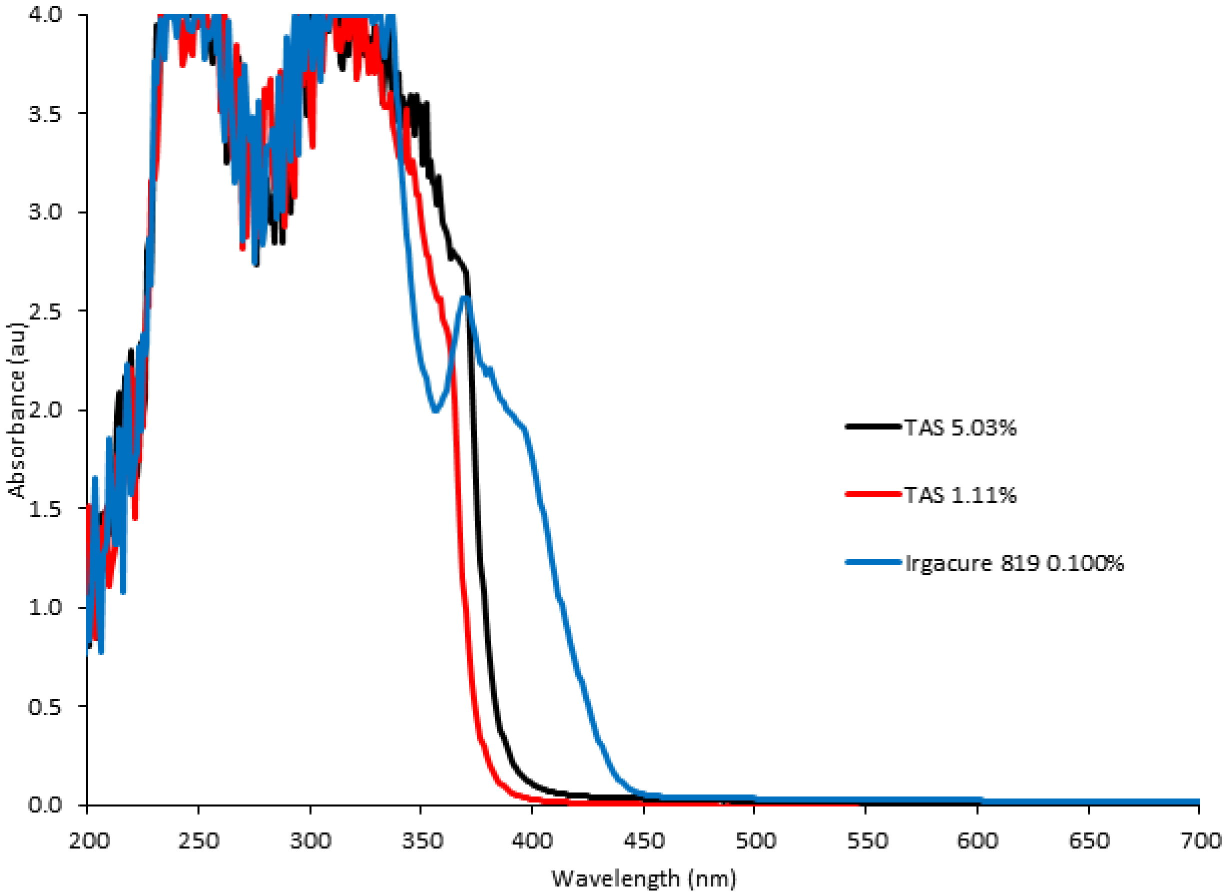

Supplementary Figure 1. UV-Vis absorption data of photoinitiators used in this study in CH_2Cl_2. Absorbance data taken at concentrations similar to those used in printing for comparison.

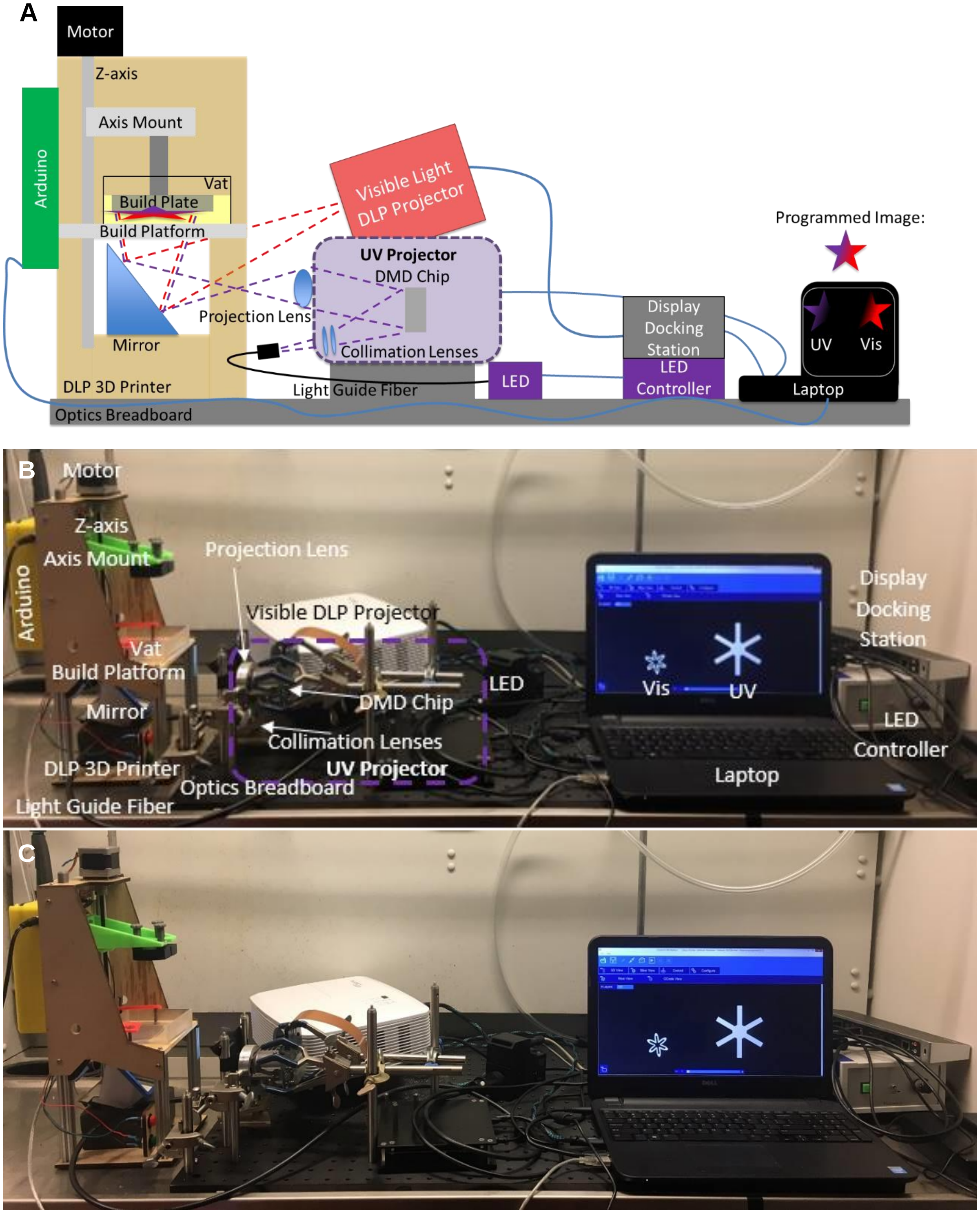

Supplementary Figure 2. (**A**) Labelled diagram of custom multimaterial DLP-AM printer setup. A laptop simultaneously controls both the visible light and UV projector, as well as the z-movement of the printer. (**B**) Labelled setup to correlate to diagram. (**C**) Physical multimaterial setup, unlabelled. Images displayed on laptop are sent to the "right" projector (UV projector, front) and the "left" projector (Optoma HD27, white projector in the back) simultaneously.

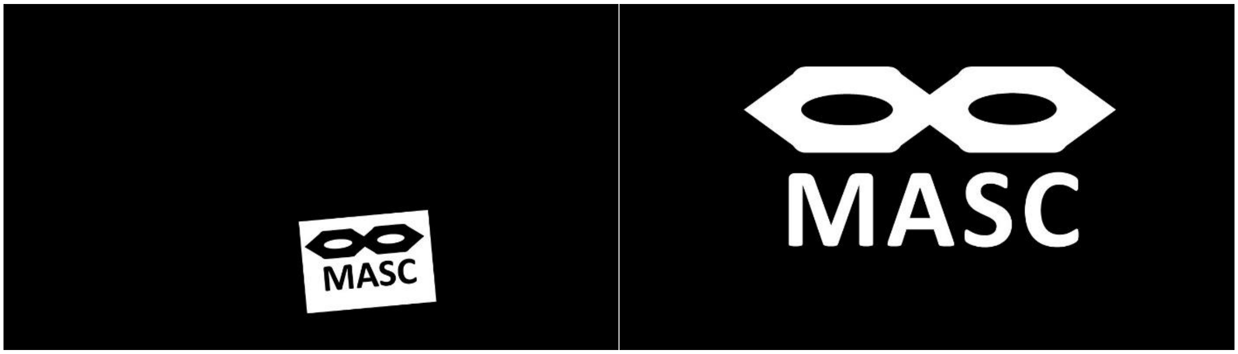

Supplementary Figure 3. Example of aligned image slices for UV projector (right) and visible light projector (left). To correct for horizontal shift, digital image for visible light projector is tilted 7 degrees.

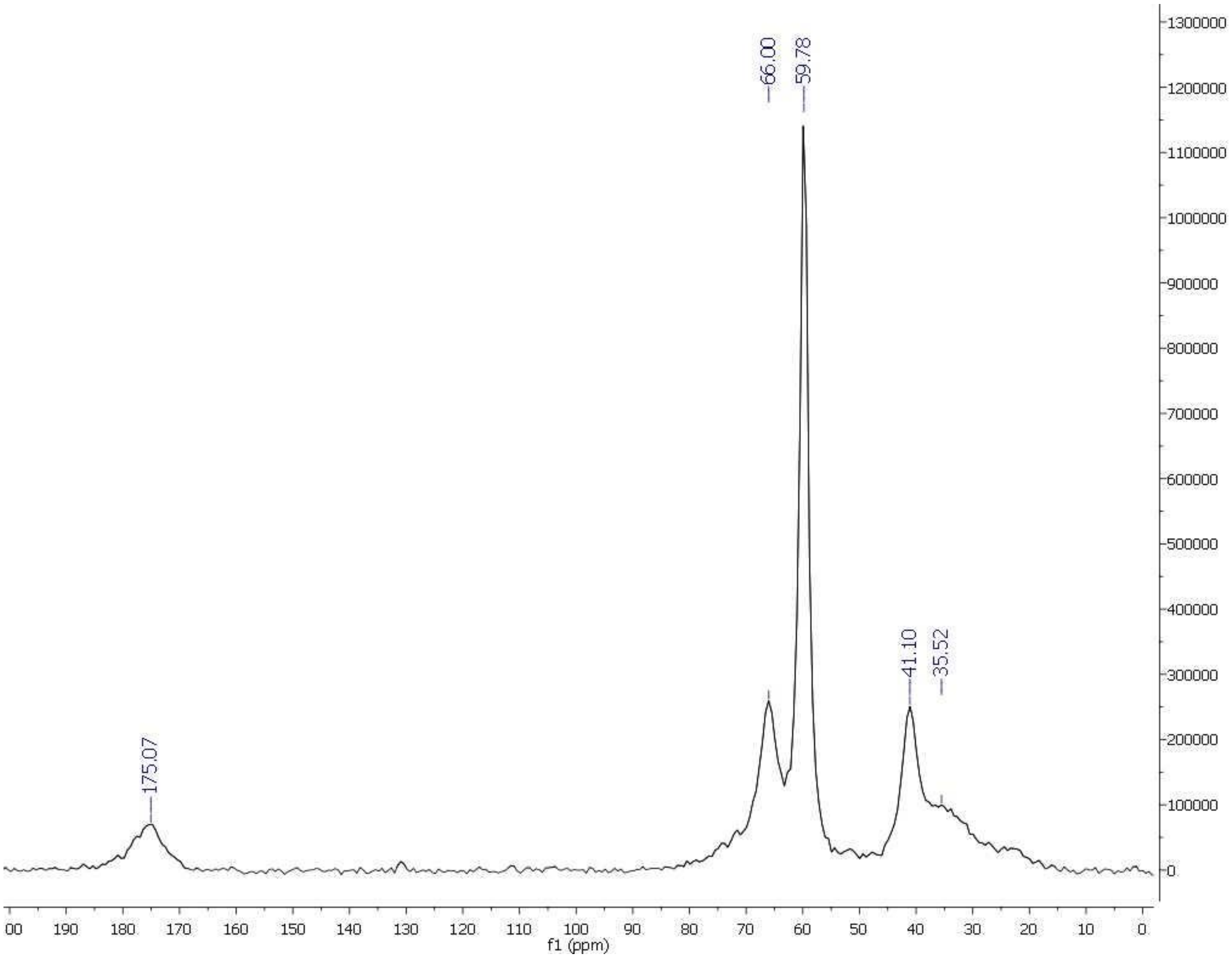

Supplementary Figure 4. CPMAS NMR spectrum of printed HEA-1 material cured with visible light with 1 min layer cure times. No visible epoxide polymer or unreacted epoxide monomer at 51 ppm.[41-43]

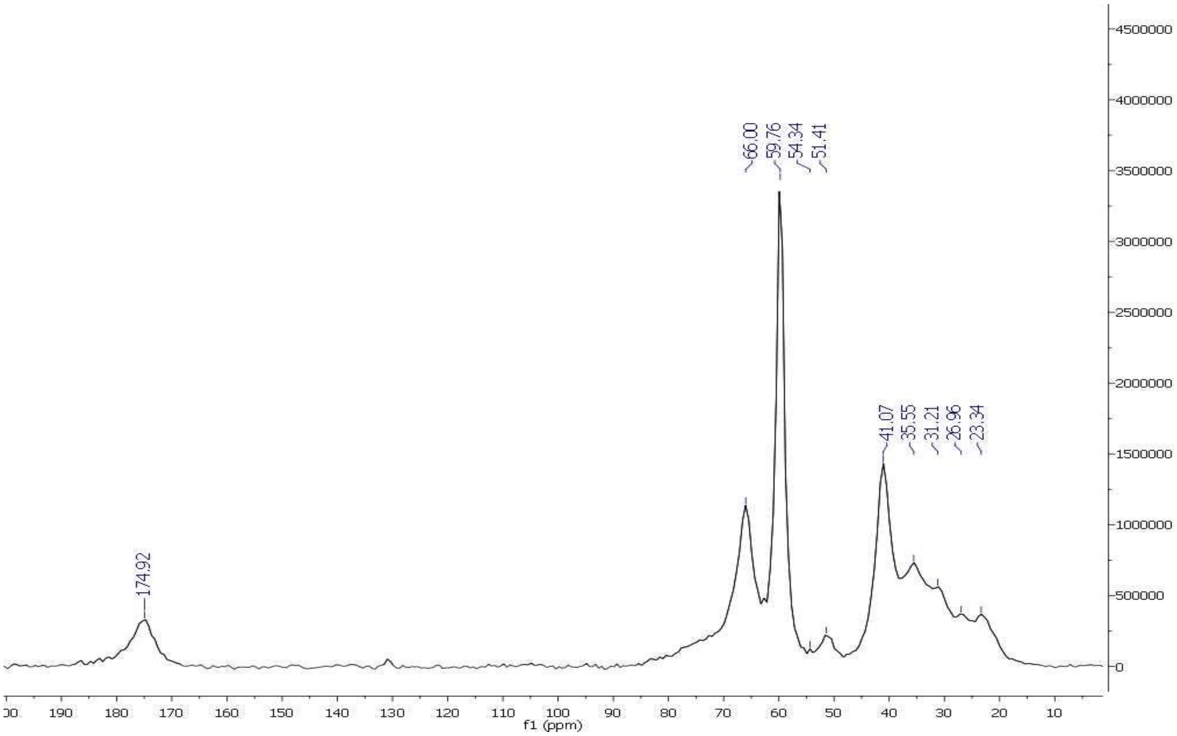

Supplementary Figure 5. CPMAS NMR spectrum of HEA-1 sample printed with UV light and 1 min layer cure times. Differences in the CH_2 region, 30-40 ppm, polymer shoulder from 70-80 ppm, and increase in polyether peak at 66 ppm support the incorporation of epoxide polymer in addition to HEA to form an epoxy-acrylate blend. Presence of unreacted epoxide peak at 51 ppm.

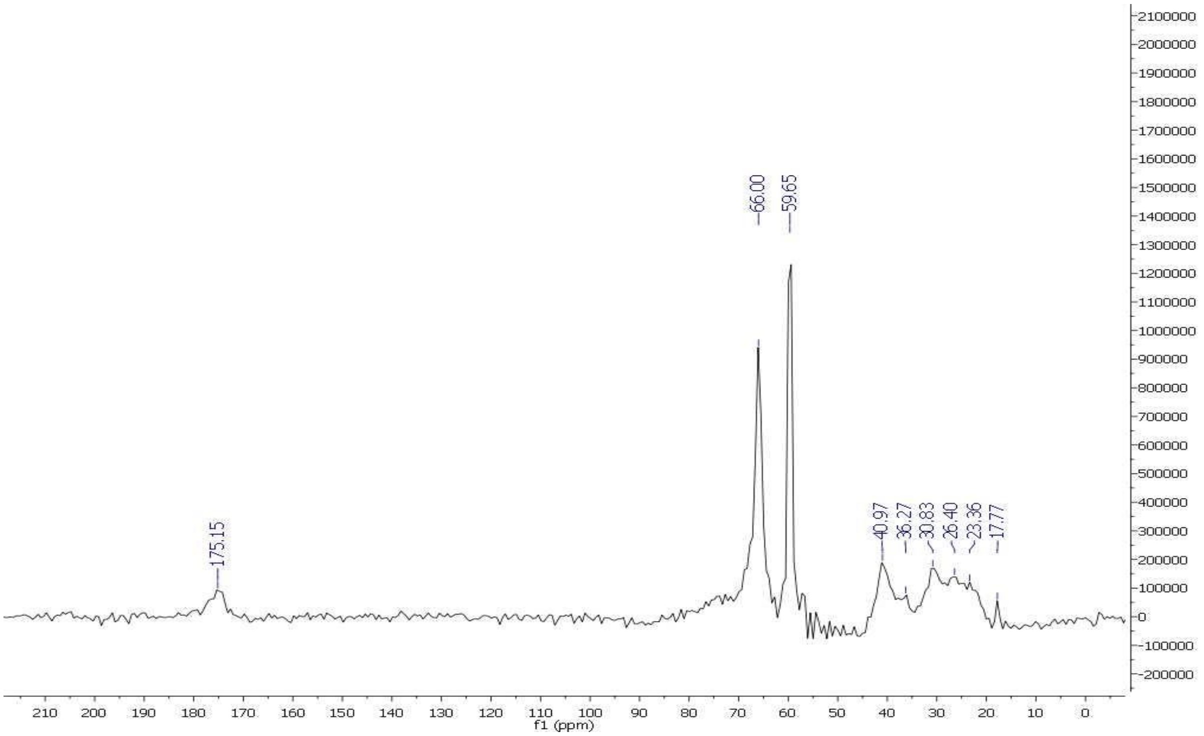

Supplementary Figure 6. CPMAS NMR spectrum of printed HEA-1 material cured with visible light using 2 min layer cure times. No visible epoxide polymer or unreacted epoxide at 51 ppm.

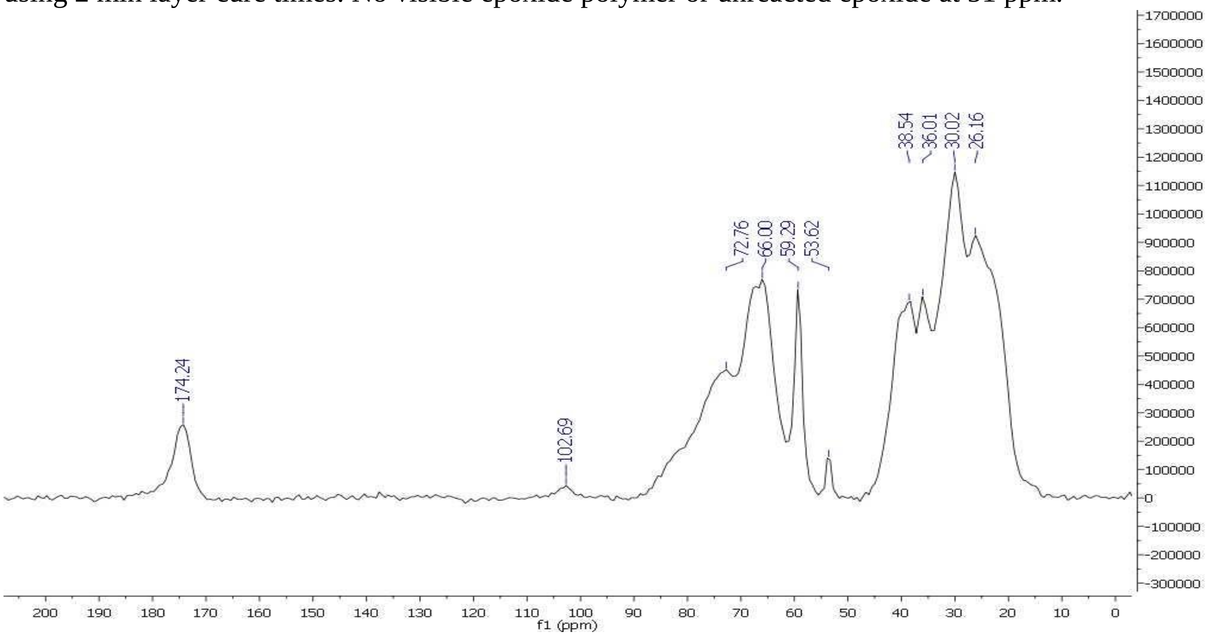

Supplementary Figure 7. CPMAS NMR spectrum of printed HEA-1 material cured with UV light using 2 min layer cure times. Differences in the CH_2 region, 30-40 ppm, polymer shoulder from 70-80 ppm, and increase in polyether peak at 66 ppm support the incorporation of epoxide polymer in addition to HEA to form an epoxy-acrylate blend. Presence of shifted unreacted epoxide peak at 54 ppm.

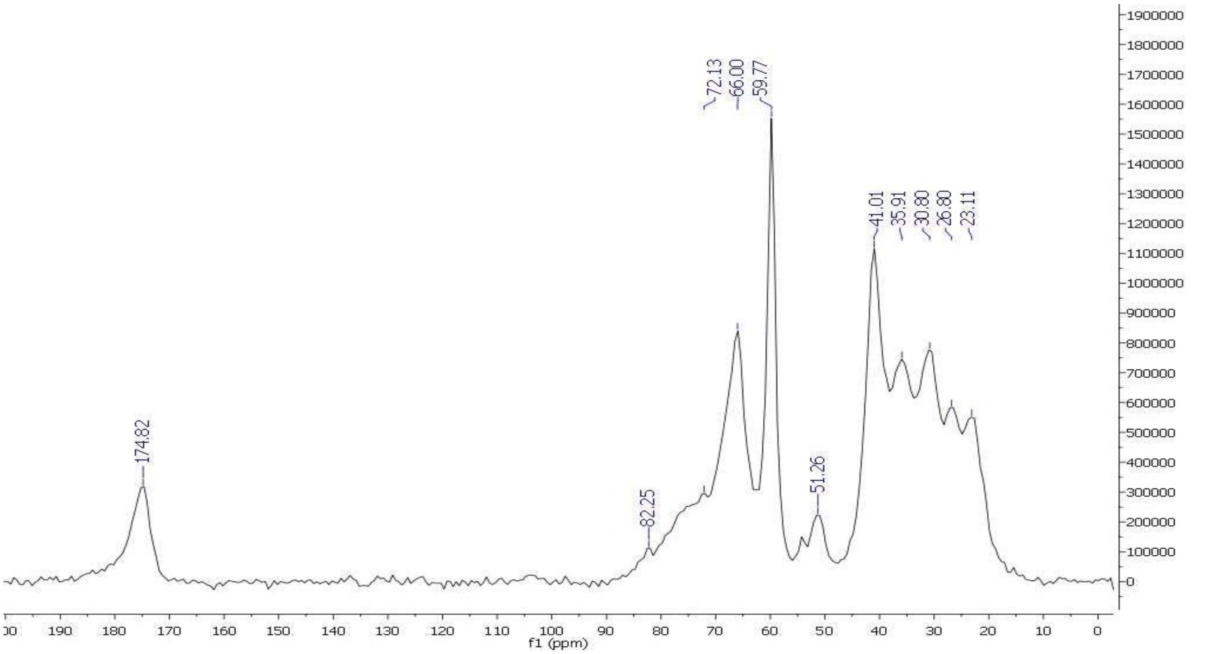

Supplementary Figure 8. CPMAS NMR of printed HEA-1 material cured with UV light using 1 min layer cure times and thermally processed for 3h at 60 ºC. Relative increases in the polymer shoulder from 70-80 ppm, the polyether peak at 66 ppm, and CH_2 peaks at 36 and 31 ppm. New shifted epoxide peak visible at 54 ppm.

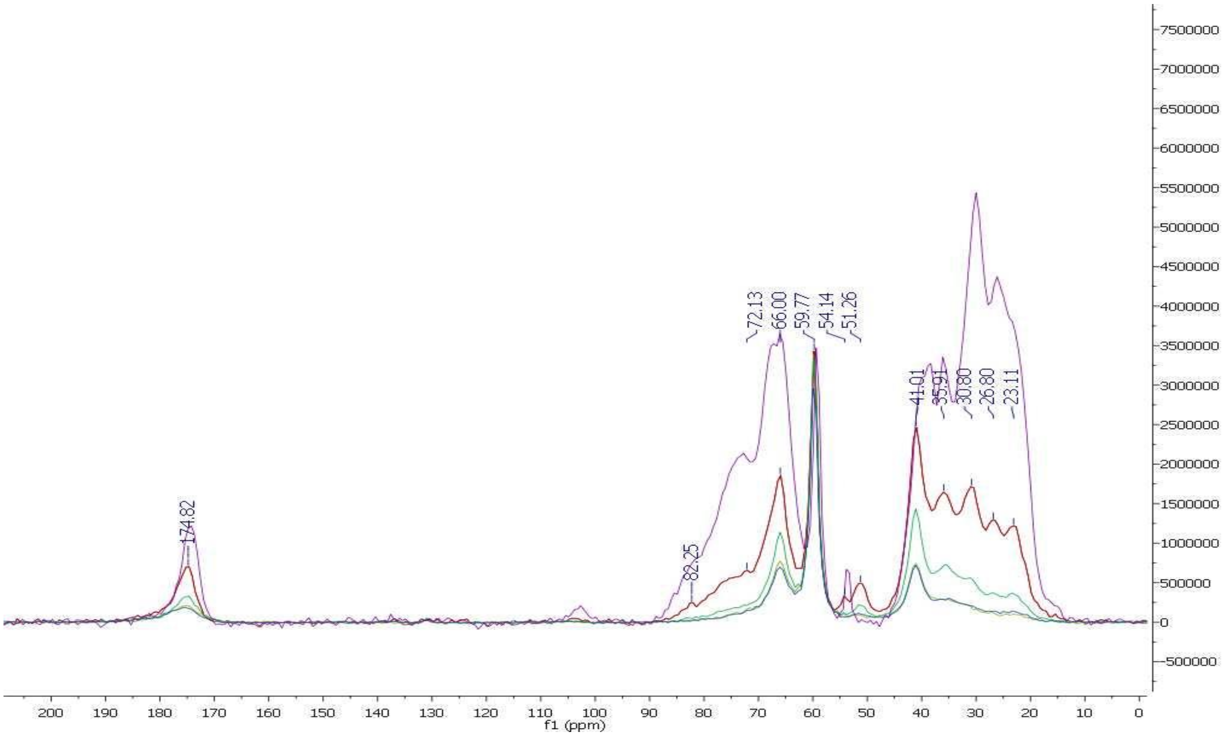

Supplementary Figure 9. Overlaid spectra from HEA-1 samples cured with visible light: 1 min layer cure time (blue), 2 min layer cure time (green), and samples cured with UV light: 1 min layer cure time (turquoise), 1 min layer cure time with 3h thermal processing (red), and 2 min layer cure times (magenta).

The lack of peaks at 51-54 ppm corresponding to unreacted epoxide in samples cured with visible light indicate that epoxide polymer was not incorporated.

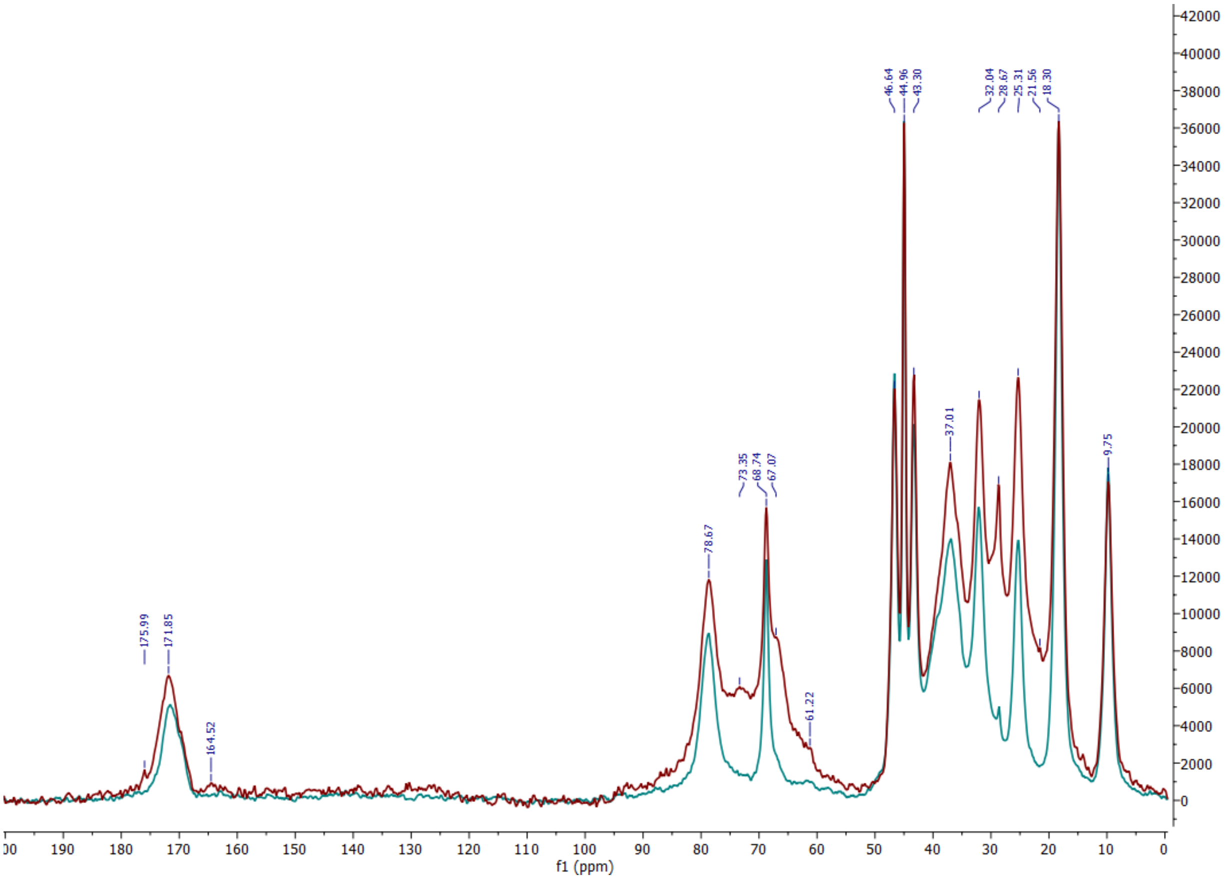

Supplementary Figure 10. Overlaid CPMAS NMR spectra of IBoA-1 samples comparing sample irradiated with UV (red) and visible light (teal). Layer cure times were 1 min with no thermal post processing. The lack of epoxide polymer peaks located at 73.35 and at the 60 -70 ppm shoulder in the sample cured with visible light, as well as the significant differences in the 20-30 ppm region indicate epoxide incorporation solely in the sample cured with UV.

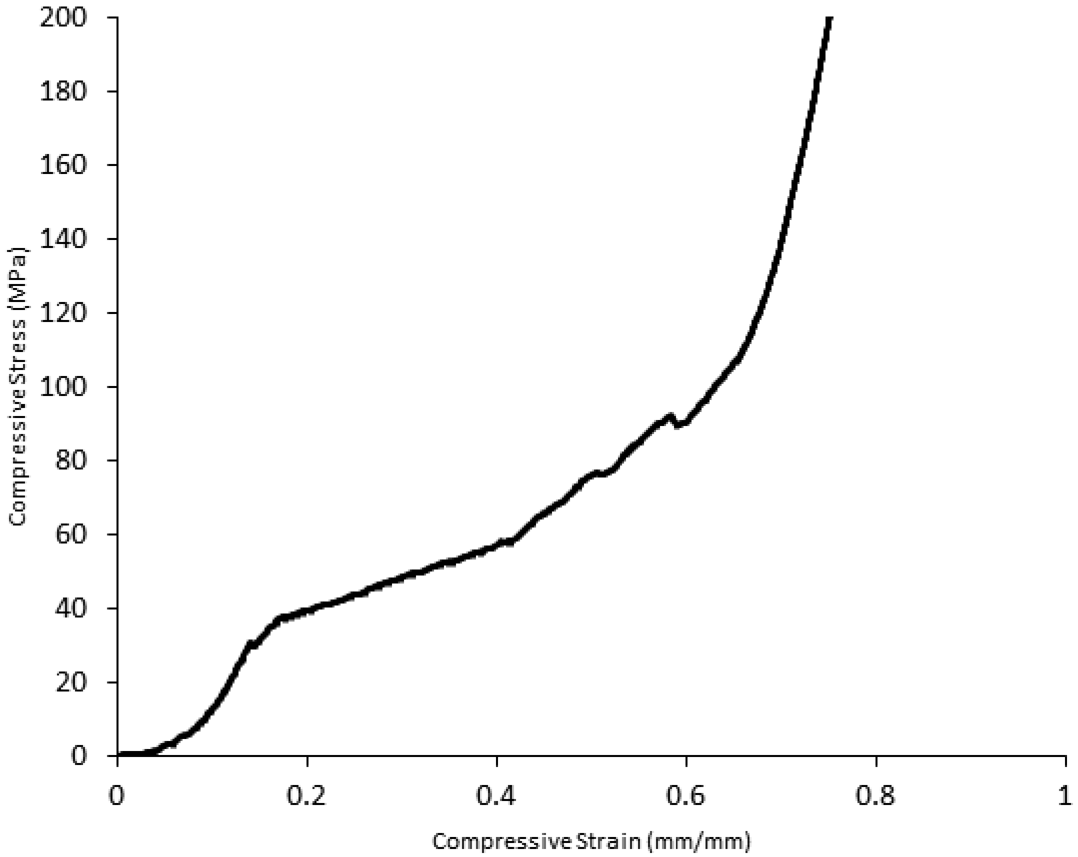

Supplementary Figure 11. Representative compressive stress-strain plot of HEA-1 sample using 250-kN load cell showing fracture at 19% compressive strain. Sample cured with UV light with 1 min layer cure times, and thermally processed for 3h at 60 °C prior to being quenched with solvent.

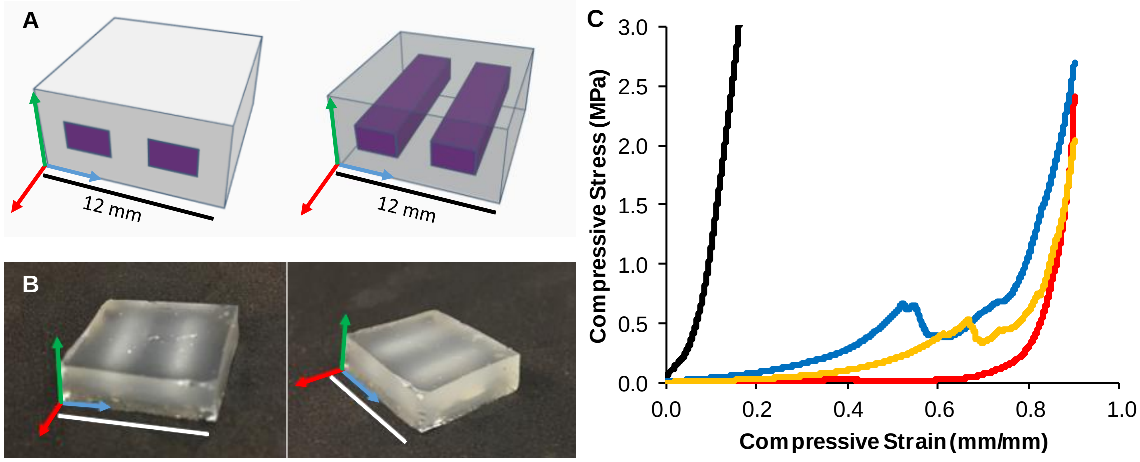

Supplementary Figure 12. Design, print, and representative stress-strain plot from compression testing of multimaterial BA-1 printed specimens. **A**) CAD models of 2-pillar BA-1 compression samples with dimensions of 12 × 12 × 6 mm^3. Purple corresponds to UV irradiation and white/transparent corresponds to visible light irradiation. **B**) Uncut printed samples are shown after swelling in spearmint oil for visual clarity of the internal pillars. **C**) Representative compressive stress-strain plots of BA-1 specimens (cut to be 12 × 4 × 4 mm^3). All samples printed with 1-min layer cure times and no thermal post-processing. Black = homogeneous sample cured with UV light. Red = homogeneous sample cured with visible light. Yellow = pillar box sample compressed along the z-axis. Blue = pillar box sample compressed along the y-axis.

Supplementary Note 1

Using the BA-1 MASC formulation, we investigated a 2-pillar design (Supplementary Figure 12) that gave better qualitative resolution of the internal pillars, in comparison with the HEA-1 system, while still enabling anisotropic effects in compressive stress-strain behavior. Multimaterial 2-pillar samples were designed and printed to have dimensions of 12 × 12 × 6 mm^3. After solvent extraction and drying, the measured dimensions were found to be 12 × 12 × 4 mm^3. The dried specimens were then cut to give test pieces that were 12 × 4 × 4 mm^3. Overall, the stress-strain behavior observed from the BA-1 samples were similar to the previous results from the 4-pillar HEA-1 samples. Once again, the differences in mechanical properties of the homogeneous samples were clearly discernable when UV (Supplementary Figure 12C, black) versus visible (Supplementary Figure 12C, red) light was used for printing. Compression of the multimaterial 2-pillar specimen along the z-axis (perpendicular to the long axis of the pillars) revealed stiffer behavior than the homogeneous soft specimen, and signs of fracture at ca. 60% compressive strain (Supplementary Figure 12C, yellow). The modulus of the specimen was greater when compressed along the y-axis (parallel with the long axis of the pillars, Supplementary Figure 12C, blue) than the z-axis. During compression along the y-axis, signs of fracture were observed at ca. 50% compressive strain.

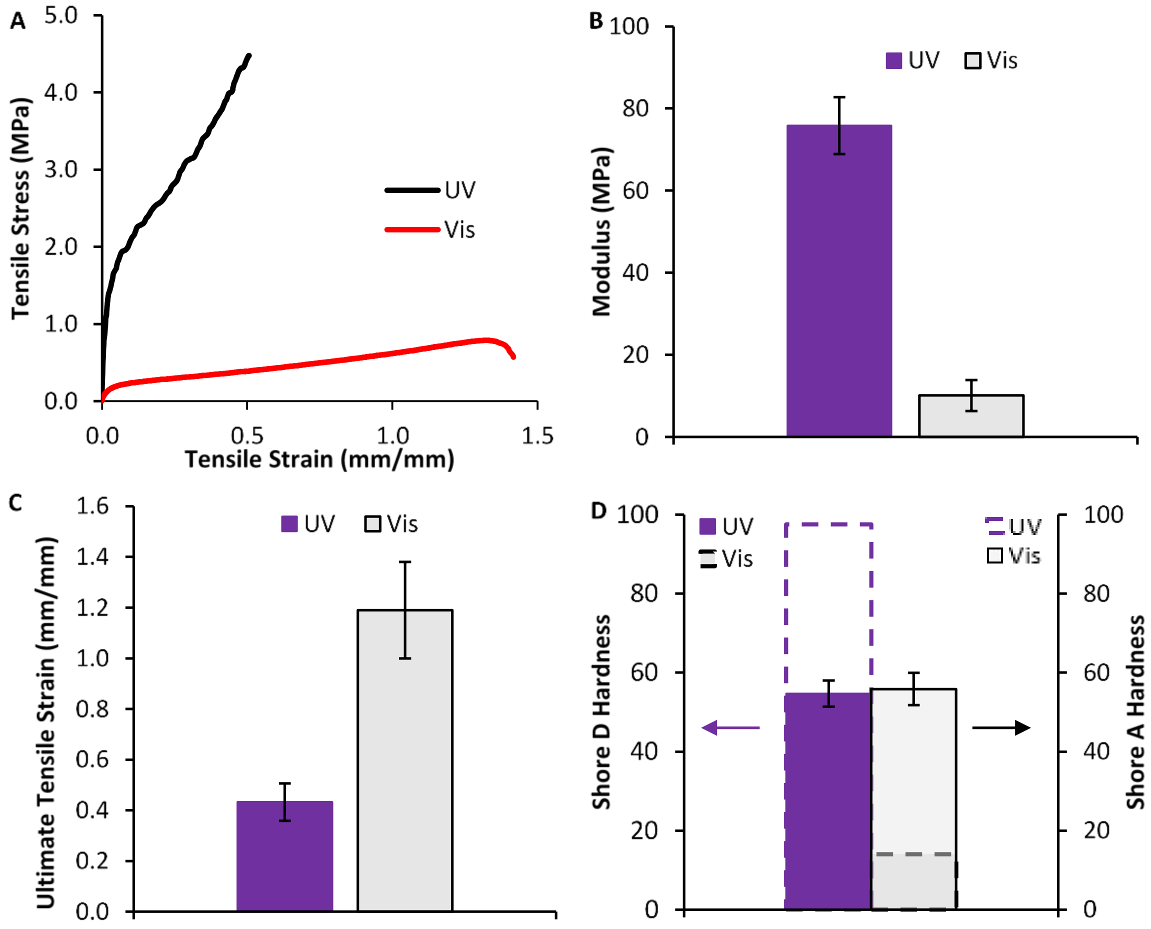

Supplementary Figure 13. Representative stress-strain plots and data from tensile testing of IBoA-1 samples. **A**) Representative stress-strain plots for uniaxial tensile test IBoA-1 specimens each printed with only one light source. To denote each specimen type, we use the light source (UV or Vis). Samples were printed with 1 min layer cure times and were immediately placed in acetone after printing (0 h of thermal post-processing). **B**) Comparison of elastic modulus; samples printed with UV (purple) or visible light (light grey) from a single vat. **C**) Comparison of ultimate tensile strain of samples printed with UV (purple) or visible light (light grey) from a single vat. **D**) Comparison of Shore hardness values; samples printed with UV (solid purple, purple arrow) were too hard to measure accurately on the Shore A hardness scale, and visible light (light grey, black arrow) were too soft to measure accurately on the Shore D hardness scale. Dotted lines for samples cured with UV (purple, theoretical Shore A hardness) and samples cured with visible light (black, theoretical Shore D hardness) denote theoretical hardness based on measured values. Samples printed from a single vat. Error bars correspond to one standard deviation.

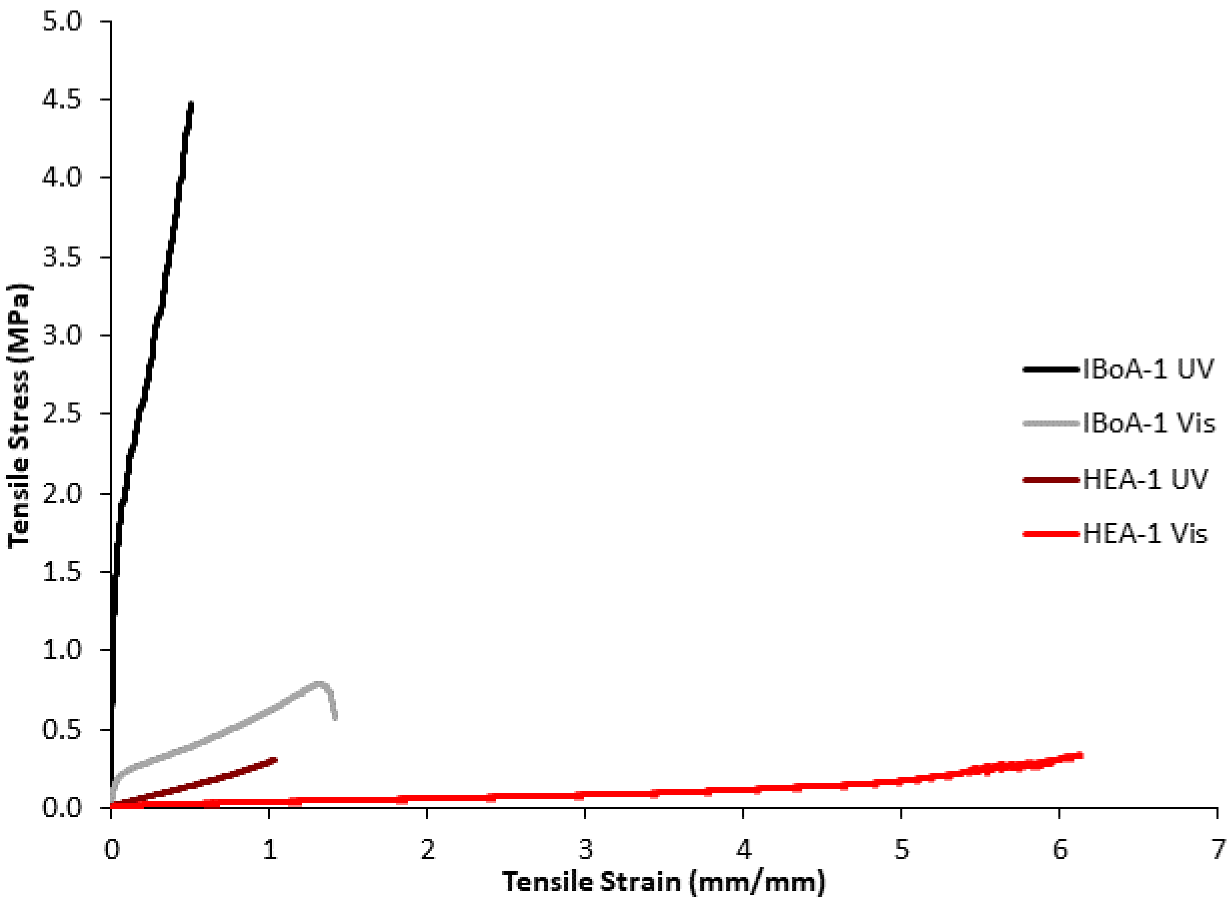

Supplementary Figure 14. Overlaid representative tensile stress-strain plots for IBoA-1 (black (UV) and grey (Vis)) and HEA-1 (dark red (UV) and red (Vis)) samples. All samples were printed with 1 min layer cure times and had no thermal post processing.

Supplementary Table 1. Gel fraction results for HEA-1, IBoA-1, and BA-1 MASC formulations.

photoresin	layer cure time (min)	gel fraction (%) cured in visible light	gel fraction (%) cured in UV light
HEA-1	1	35	52
HEA-1	2	37	69
IBoA-1	1	46	64
BA-1	1	23	50

Supplementary Table 2. Swelling ratios for samples printed with 1 min layer times and no thermal processing.[a]

photoresin	light source	mass ratio	volume ratio
HEA-1	UV	1.30 ± 0.10	0.92 ± 0.22
HEA-1	Vis	2.92 ± 0.70	4.27 ± 1.01
HEA-1	UV then Vis[b]	2.02 ± 0.10	1.90 ± 0.17
BA-1	UV	2.25 ± 0.45	2.17 ± 1.18
BA-1	Vis	3.91 ± 0.21	4.30 ± 1.58

[a]Swelling conducted in deionized water for HEA-1 materials and toluene for BA-1 materials. Disc-shaped samples were used having an as-designed diameter of 12 mm and 15 layers (each 100 µm) in height. Layer cure time = 1 min. Post-processing for HEA-1: swelling in water followed by acetone wash. Post-processing for BA-1: swelling in CH_2Cl_2 followed by acetone wash. Monomer extraction was considered complete when the change in dry weight between swelling was less than 3%. For swelling, HEA-1 specimens were submerged in deionized water for 3 h; BA-1 specimens were submerged in toluene for 2 h. Swelling ratios are an average of 3 experiments, errors = standard deviation.; [b] Samples printed UV then Vis were printed with 8 layers of UV followed by 8 layers of visible light. Values indicate representations of mean values ± standard deviations (n=3).

Supplementary Table 3. Swelling ratios of HEA-1 samples with 1 min layer cure times and 3 h thermal processing at 60 °C.[a]

light source	mass ratio	volume ratio
UV	0.42 ± 0.05	0.46 ± 0.15
Vis	3.12 ± 0.19	3.23 ± 0.17
UV then Vis	1.06 ± 0.30	0.70 ± 0.27

[a]Based on HEA/EPOX (1 min layer cure times, no thermal post-cure), a UV then Vis sample has a swelling ratio roughly halfway between its visible light and UV cured homogeneous counterparts, as expected. With thermal processing, the swelling ratio is now 60% of the expected value (cf. Supplementary Table 1). Values indicate representations of mean values ± standard deviations (n=3).

Supplementary Movie 1. (left) Video of differential swelling-induced 4D actuation of HEA-1 sea star in water over the span of 2 h. View from the top (top left) and side (bottom left) of the swelling chamber. Both videos taken at the same time. (right) Video of differential swelling-induced 4D actuation of BA-1 sea star in toluene over the span of 2 h. View from the top (top right) and side (bottom right) of the swelling chamber. Both videos taken at the same time.
