# Supplementary material for: Multimaterial actinic spatial control 3D and 4D printing
Source: Nat Commun. 2019 Feb 15;10:791. doi: 10.1038/s41467-019-08639-7 (PMC6377643; doi:10.1038/s41467-019-08639-7)
Supplement: Supplementary file 2 — Description of Additional Supplementary Files [file 41467_2019_8639_MOESM2_ESM.pdf]

Description of Additional Supplementary Files

File Name: Supplementary Movie 1

Description: (left) Video of differential swelling-induced 4D actuation of HEA-1 sea star in water over the span of 2 h. View from the top (top left) and side (bottom left) of the swelling chamber. Both videos taken at the same time. (right) Video of differential swelling-induced 4D actuation of BA-1 sea star in toluene over the span of 2 h. View from the top (top right) and side (bottom right) of the swelling chamber. Both videos taken at the same time.
